# Glutamine uptake and utilization of human mesenchymal glioblastoma in orthotopic mouse model

**DOI:** 10.1186/s40170-020-00215-8

**Published:** 2020-08-10

**Authors:** Kristell Oizel, Chendong Yang, Ophelie Renoult, Fabien Gautier, Quyen N. Do, Noemie Joalland, Xiaofei Gao, Bookyung Ko, François Vallette, Woo-Ping Ge, François Paris, Ralph J. DeBerardinis, Claire Pecqueur

**Affiliations:** 1 Children’s Research Institute, UT Southwestern Medical Center, Dallas, TX 75390 USA; 2 Université de Nantes, CNRS, INSERM, CRCINA, Nantes, France; 3 Institut de Cancérologie de l’Ouest, Saint-Herblain, France; 4 Department of Radiology, UT Southwestern Medical Center, 5323 Harry Hines Blvd, Dallas, TX 75390-9061 USA; 5 LabEx IGO “Immunotherapy, Graft, Oncology”, Nantes, France; 6 Department of Pediatrics, Neuroscience, Neurology & Neurotherapeutics, University of Texas Southwestern Medical Center, Dallas, TX 75390 USA; 7 Howard Hughes Medical Institute, Chevy Chase, USA

**Keywords:** Glioblastoma, Metabolism, Molecular subtype, Mesenchymal, Glutamine, Human primary cells, Orthotopic model

## Abstract

**Background:**

Glioblastoma (GBM) are highly heterogeneous on the cellular and molecular basis. It has been proposed that glutamine metabolism of primary cells established from human tumors discriminates aggressive mesenchymal GBM subtype to other subtypes.

**Methods:**

To study glutamine metabolism in vivo, we used a human orthotopic mouse model for GBM. Tumors evolving from the implanted primary GBM cells expressing different molecular signatures were analyzed using mass spectrometry for their metabolite pools and enrichment in carbon 13 (^13^C) after ^13^C-glutamine infusion.

**Results:**

Our results showed that mesenchymal GBM tumors displayed increased glutamine uptake and utilization compared to both control brain tissue and other GBM subtypes. Furthermore, both glutamine synthetase and transglutaminase-2 were expressed accordingly to GBM metabolic phenotypes.

**Conclusion:**

Thus, our results outline the specific enhanced glutamine flux in vivo of the aggressive mesenchymal GBM subtype.

## Introduction

With an incidence of 5 per 100,000, glioblastoma (GBM), grade IV glioma, is the most frequent primary brain tumor in adults. Its prognosis is dismal, with a 5-year survival under 5% and a mean survival of 15 months despite aggressive treatment. These treatments include surgery followed by concomitant radio- and chemotherapy with temozolomide. Unfortunately, no significant improvement in the therapy has been made since 2009 with the inclusion of temozolomide (TMZ) as a radiosensitizer in the clinical protocol [1]. Many factors could explain failure of current therapies. Besides being highly infiltrative, essentially eliminating the possibility of complete resection, GBM display a very heterogeneous profile on a cellular and molecular basis leading to different patient responses to identical treatment [2]. In the past 10 years, 4 molecular subtypes (mesenchymal, classical, neural, and proneural) have been established based on genetic and molecular alterations as well as patient’s prognosis [3, 4]. However, a recent study with extensive gene expression profiling both at the whole tumor level and individual tumor cells highlights 2 main tumor-intrinsic transcriptional subtypes, the mesenchymal and the non-mesenchymal (defined in our study as CNP, for classical, neural, and proneural) [5].

From the cellular heterogeneity point of view, the presence of cancer stem cells (CSCs) inside the tumor could play a role in the resistance through their low proliferative profile and their enhanced DNA repair machinery [6]. Furthermore, CSCs generate cellular heterogeneity by installing a differentiation hierarchy leading to various distinct cell types present within the tumor [7]. Unfortunately, the efficacy of CSC targeting has been difficult to study due to the limited characterization of CSC markers. Several markers, such as CD133, CD44, CD166, CD24, and ALDH1 activity, have proven useful for prospective isolation of CSCs in multiple solid tumors [8]. However, CSC marker expression is not uniform between tumor types. For instance, while CD133 has been used as a marker to identify CSCs in GBM, it is not expressed by CSCs belonging to the mesenchymal subtype [9].

We have recently shown that human primary cultures derived from GBM patient after surgery capture both the molecular and the cellular heterogeneity, and as such are powerful tools for investigating tumor biology. Using these cells, we showed that in vitro, the molecular signature mirrors a metabolic signature. While the CNP subtype strongly relied on glucose, survival and proliferation of the mesenchymal GBM subtype were strongly dependent on glutamine. However, recent studies have challenged tumor reliance on glutamine in vivo when GBM cells used glucose rather than glutamine to produce energy and to provide an anaplerotic flux for the TCA cycle in 3 different primary human GBM transplanted orthotopically in mice [10]. Moreover, cells derived from those tumors did not require glutamine to sustain viability and proliferation when cultured ex vivo. Altogether, these studies prompted us to better clarify on the role of glutamine metabolism in mesenchymal GBM cells in such integrated tumor models.

Here, using an orthotopic murine model deriving from either mesenchymal or CNP GBM subtypes, we provide evidence of enhanced glutamine uptake and utilization in the mesenchymal GBM in vivo.

## Materials and methods

Unless stated otherwise, all cell culture material was obtained from Life Technologies (Cergy Pontoise, France) and chemicals were from Sigma-Aldrich (St. Louis, MO, USA).

### Human GBM tumor cells

Primary GBM cultures were derived after mechanical dissociation from high-grade glioma operated on 4 patients. All procedures involving human participants were in accordance with the ethical standards of the ethic national research committee and with the 1964 Helsinki Declaration and its later amendments or comparable ethical standards. Informed consent was obtained from all individual participants included in this study. Primary GBM cells were cultured in defined medium (DMEM/HAM-F12, 2 mM l-glutamine, N2 and B27 supplement, 2 μg/ml heparin, 20 ng/ml EGF and 25 ng/ml bFGF, 100 U/ml penicillin, and 100 μg/ml streptomycin). All the experiments with primary GBM cells were performed at early passages (< 10 passages). When indicated, cells were treated with CB839 (20 μM) or EGCG (110 μM) for the indicated time. Cells were checked for mycoplasma regularly. Molecular signature, as well as gene amplification or loss, was assessed using the GEO database previously deposited under accession number (GSE83626) [9], either by unsupervised hierarchical clustering or by Gene Set Enrichment Analysis (GSEA) on R using fgsea package [11].

### Western blots and immunohistochemistry

Fifty micrograms of cell lysates was used for western blot analysis [12]. Primary antibodies and secondary antibodies coupled to HRP were used according to the manufacturer’s recommendations. For immunochemistry, paraffin-embedded specimens were fixed in 4% PFA and then stained with a rabbit anti-human MHC class I (clone EPR1394Y; Abcam).

### Seahorse analysis

Mitochondrial oxygen consumption (OCR) and extracellular acidification rate (ECAR) were measured in non-buffered medium containing 0.2 mmol/l cystine supplemented with glucose (5 mmol/l), pyruvate (1 mmol/l), and glutamine (2 mmol/l) using an XF24 Analyzer (Seahorse Bioscience). Specific mitochondrial respiration fueled with either glucose (**∆**OCR_GLC_) or glutamine (**∆**OCR_GLN_) was determined as previously described [9] by the difference of the mean of the 3 values of OCR in the absence of substrate and the mean of the 4 values of OCR after injection of the substrate. Glucose was injected to a final concentration of 10 mmol/l and glutamine 2 mmol/l.

### Orthotopic injections of human primary GBM cells in NSG mice

All animal experiments were carried out in accordance with protocols approved by the Institutional Animal Care and Use Committee at the University of Texas Southwestern Medical Center, and to French institutional guidelines (agreement # 00186.02; regional ethics committee of the Pays de la Loire, France). For global metabolite enrichment, orthotopic injections of 10^4^ human GBM cells were performed using a stereotactic frame (Stoelting) at 2 mm on the right of the medial suture and 0.5 mm in front of the bregma, with a depth of 2.5 mm. Animals were observed daily and euthanized when characteristic symptoms occurred, such as reduced mobility and significant weight loss. For ^13^C enrichment infusions, 10^4^ tumor cells in suspension were transplanted in mouse brains at the same coordinates via a glass micropipette (WIRETROL, DRUMMOND®) with a 50-μm tip generated by a Micropipette Puller (P-97, Sutter Instrument Co.). Tumor growth was regularly monitored using a 1-T Desktop magnetic resonance scanner (M2 Compact, Aspect Imaging, Shoham, Israel) and a mouse head coil when characteristic symptoms started to occur such as weight loss. The general T1-weighted and T2-weighted imaging was performed with a spin echo (TR/TE = 326/13 ms) and a fast spin echo (TR/TE = 2500/80 ms) sequence, respectively (prone position). When the diameter of the tumor reached 3 mm, mice were infused with ^13^C_5_ glutamine (99% enrichment; Cambridge Isotope Laboratories, Andover, MA) through the jugular vein. A bolus of 187 mg/kg of labeled glutamine diluted in 0.2 ml saline was first injected within 1 min, and then, 5 mg/kg/min was perfused during 5 h.

Blood was collected before ^13^C_5_ glutamine perfusion then at different time points until the end of the perfusion, and the plasma was used to determine the enrichment in ^13^C_5_ glutamine. At the end of the perfusion, mice were decapitated and brain and liver tissues were collected. Brain tumor and contralateral tissues were rapidly dissected under a microscope, weighed, transferred in 1 ml of 80% methanol solution, and stored at − 80 °C before further analysis.

### Metabolite extraction and measurement of ^13^C fractional enrichments in tissue and cell samples

Snap-frozen tissues collected from different tumor-bearing mice, or cell samples were homogenized in ice-cold methanol. Metabolite extraction for LC-MS/MS analysis was prepared as previously described [13]. Peaks were normalized against the total ion count and tissue weight. For ^13^C enrichments, homogenates were subjected to three rapid freeze-thaw cycles by transferring them from liquid nitrogen to a 37 °C water bath. Samples were centrifuged at 13,000*g* at 4 °C for 15 min, and the supernatant transferred to a screw-topped glass tube with 50 nM of sodium-2-oxobutyrate then completely evaporated at 42 °C under blown air. Evaporated samples were re-suspended in 30 μl pyridine containing methoxyamine (10 mg/ml). After 10 min at 70 °C, 70 μl of MTBSTFA reagent was added and heated at 70 °C for 1 h. GC-MS was performed using an Agilent 6890N Gas Chromatograph coupled to an Agilent 5973 Mass Selective Detector (Agilent Technologies, Santa Clara, CA). One microliter of each standard or sample was injected and analyzed in scan mode.

### Measurement of ^13^C fractional enrichments in blood

Blood samples were processed to measure ^13^C_5_ enrichment in glutamine by gas chromatography-mass spectrometry (GC-MS), as previously described [10]. A 3-point standard curve was prepared by mixing unenriched glutamine with ^13^C_5_ glutamine such that 0%, 50%, or 100% of glutamine was ^13^C labeled. GC-MS was performed using an Agilent 6890N Gas Chromatograph coupled to an Agilent 5973 Mass Selective Detector (Agilent Technologies, Santa Clara, CA). One microliter of each standard or sample was injected and analyzed in scan mode. Fragment ions of *m/z* 258 (unenriched) and 263 (enriched) ^13^C_5_ glutamine were quantified for both standard and experimental samples. Linear regression was used to calculate the enrichment of each plasma sample.

### Statistical analysis

Data were analyzed, and statistical analyses were performed using GraphPad Prism 6.00 (GraphPad Software, San Diego, CA, USA). Data points are expressed as mean ± SD unless otherwise indicated. For statistical analyses, results are compared to the CTR group unless stated otherwise: *p < 0.05, **p < 0.01, and ***p < 0.001. Hierarchical clustering was realized using XLSTAT software.

## Results

### Metabolic and molecular signatures of human GBM primary cultures in vitro

**Fig. 1:**
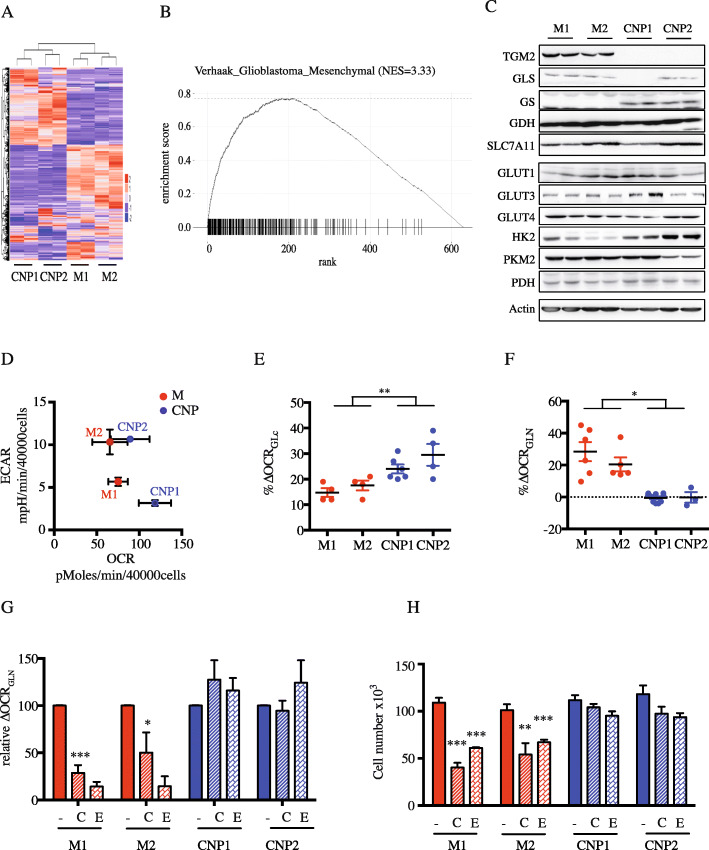
Human primary culture characterization. **a** Heat map of unsupervised hierarchical classification. **b** Molecular subtypes assigned by GSEA. **c** Protein abundance of glycolytic and glutaminolytic enzymes: transglutaminase 2 (TGM2), glutaminase (GLS), glutamine synthetase (GS), glutamate dehydrogenase (GDH), Cystine/glutamate antiporter XCT/SLC7A11, glucose transporter (GLUT), hexokinase 2 (HK2), isoform M2 of pyruvate kinase (PKM2), and pyruvate dehydrogenase (PDH). Actin was used as a loading control. **d** Global metabolism using the Seahorse technology. OCR (oxygen consumption rate) and ECAR (extracellular acidification rate) were measured (*n* > 3 for each primary cultures). **e**, **f** Mitochondrial respiration rate based on glutamine (∆OCR_GLN_; **e**) or glucose (∆OCR_GLC_; **f**). Results are presented as mean ± SEM, *n* = 5 for each primary cultures. *p < 0.05; **p < 0.01. **g** Inhibition of mitochondrial respiration rate based on glutamine 5 h after addition of glutaminase and GDH inhibitors, CB839 (C) and EGCG (E), respectively. Results are presented as mean ± SEM, *n* = 3 for each primary cultures. *p < 0.05; ***p < 0.001. **h** Proliferation of primary GBM cells after 72 h in the presence of glutaminase and GDH inhibitors, CB839 (C) and EGCG (E), respectively. Results are presented as mean ± SEM, *n* = 3 for each primary cultures. **p < 0.01; ***p < 0.001

Tumor samples from 4 different patients were dissociated and cultured in defined media in order to maintain their original molecular and cellular heterogeneity. As shown in Fig. 1a, unsupervised hierarchical transcriptomic analysis clearly identified 2 molecular subgroups. Two primary cultures displayed a mesenchymal signature (M1 and M2) as shown by GSEA profiling (Fig. 1b), in contrast to the other primary cultures labeled here as CNP1 and CNP2, respectively, as previously described [9]. All primary cultures expressed PTEN but displayed the genetic loss of INK4a/ARF locus (Supplementary Table 1). Of note, CNP1 also exhibited genetic EGFR and PDGFR amplification. We next examined the expression of several enzymes involved either in glycolysis or in glutamine metabolism (Fig. 1c). For most enzymes, we did not observe any difference in their expression. As expected, transglutaminase 2 (TGM2) was exclusively expressed in mesenchymal GBM cells. Surprisingly, glutamine synthetase (GS) expression was restricted to CNP cells. Metabolic analysis performed using the Seahorse technology, measuring respectively mitochondrial respiration (OCR) and glycolysis through extracellular acidification (ECAR), did not show significant difference between mesenchymal and CNP cells (Fig. 1d). However, a finer analysis of the substrates fueling mitochondrial respiration clearly distinguished the 2 subtypes (Fig. 1e, f). All primary cells used glucose to sustain their oxidative metabolism, but CNP cells demonstrated modestly enhanced glucose oxidation compared to mesenchymal cells. More impressively, mesenchymal cells used glutamine to sustain oxidative phosphorylation to a much greater extent than CNP cells. To determine whether glutamine metabolism drives mesenchymal GBM cell proliferation, primary GBM cells were cultured in the presence of CB839 and EGCG. These 2 molecules have been previously described as inhibitors of glutamine metabolism, targeting glutaminase and glutamate dehydrogenase (GDH), respectively. As expected, each compound significantly reduced glutamine-based mitochondrial respiration rate in mesenchymal cultures without affecting CNP mitochondrial respiration (Fig. 1g). We then determined primary GBM cell proliferation in the presence of these inhibitors. Each inhibitor significantly reduced the proliferation of mesenchymal GBM cultures (Fig. 1g). Importantly, these inhibitors did not affect the proliferation of CNP cultures. Altogether, our results clearly illustrate a different metabolic profile between mesenchymal and CNP cultures in vitro.

### Mesenchymal human orthotopic tumors (HOT) are enriched for glutamine and glutamine-derived metabolites

**Fig. 2:**
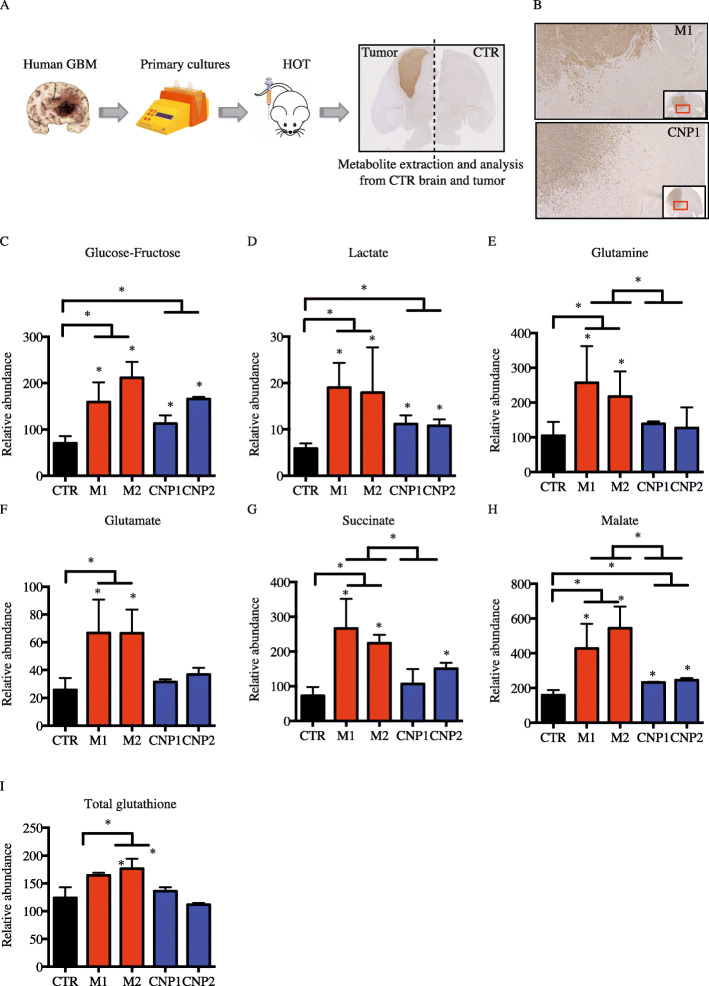
Metabolic profile in human orthotopic tumors (HOT) and control brain. **a** The experimental protocol. **b** IHC analysis of representative mouse HOT for each molecular subtype derived from parental primary cultures (M1, CNP1) stained with MHC-I antibody. **c**–**i** Relative abundance of glucose-fructose (**c**), lactate (**d**), glutamine (**e**), glutamate (**f**), succinate (**g**), malate (**h**), and total glutathione (**i**) from 3 independent samples for CTR brain and each tumor subtype. Results are presented as mean ± SEM. Results are compared to CTR brain and between groups using multiple *t* test analysis (*p < 0.05)

To investigate tumor metabolism in vivo, cells from human primary tumor were implanted into the striatum of one brain hemisphere (see details in the “Materials and methods” section) of NOD-SCID-gamma (NSG) mice (Fig. 2a). After 3 to 5 months, the mice presented symptoms as the result of an expanding tumor mass. Brains were then collected, split between the tumor and the contralateral hemispheres (defined here as CTR) (Fig. 2a), and immediately frozen for metabolic studies. Histological analysis using anti-MHC-I antibodies demonstrated that despite the invasive features of primary GBM cells, most of the tumor mass resided within one hemisphere (Fig. 2b). We then used liquid chromatography-tandem mass spectrometry (LC-MS/MS) to determine the relative quantities of 105 metabolites extracted from each specimen. As expected, glycolytic products and intermediates, such as glucose, glucose 6-phosphate (G6P), and lactate, were significantly enriched in tumor with no significant differences between subtypes (Fig. 2c, d and Supplementary S1). Ribose 5P, an intermediate of the pentose phosphate pathway, was also significantly more abundant in tumors compared to CTR brain (Supplementary S1). We next examined closely the relative enrichment of glutamine and glutamine-derived metabolites from each specimen. Interestingly, our results showed higher glutamine abundance in M1 and M2 tumors but not in CNP1 and CNP2 tumors (Fig. 2e). Glutamate, which can be derived from glutamine through the activity of glutaminase, was also more enriched in M1 and M2 tumors compared to either CTR brain or CNP tumors (Fig. 2f). Once glutamine is converted to glutamate, glutamate dehydrogenase (GDH) or transaminases convert glutamate to α-ketoglutarate, entry point into the tricarboxylic cycle (TCA) where it will be converted into succinate and malate. Interestingly, whereas there was a global increase of succinate and malate in all HOT, mesenchymal tumors displayed higher levels of succinate and malate compared to CNP tumors (Fig. 2g, h). Finally, the synthesis of glutathione, a tripeptide of glutamate, cysteine, and glycine, is usually dependent on glutamine metabolism in cells. In agreement with our previous results, glutathione was more abundant in mesenchymal tumors compared to CTR brain or CNP tumors (Fig. 2i). Altogether, our results showed that mesenchymal HOT were enriched in glutamine and glutamine-derived metabolites compared to either CTR brain or CNP tumor.

### Increased ^13^C-glutamine uptake in mesenchymal HOT

**Fig. 3:**
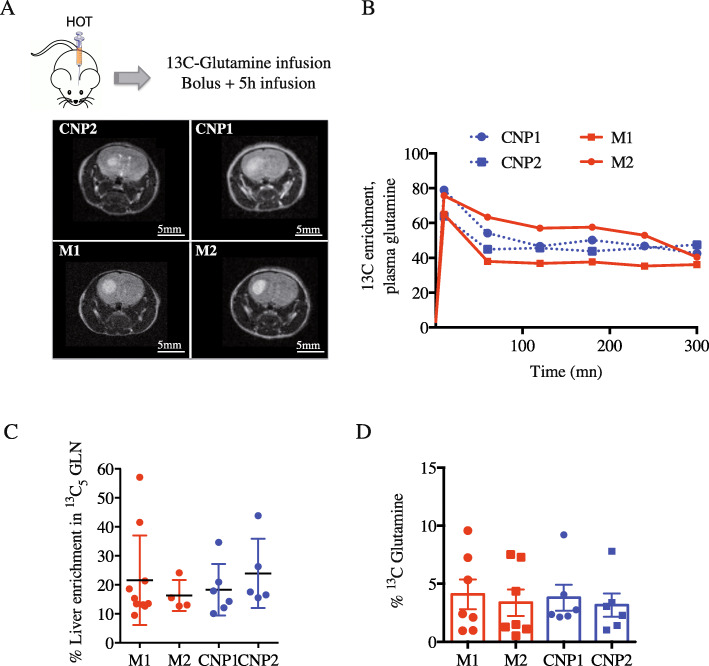
^13^C-glutamine infusions in HOT-bearing mice. **a** MRI of representative mouse HOT derived from each parental primary culture (M1, M2, CNP1, CNP2). **b** HOT-bearing mice derived from each parental primary culture (M1, M2, CNP1, CNP2) were infused with ^13^C_5_-glutamine for the indicated times. The time course shows representative ^13^C-glutamine enrichment in plasma (%). All mice received a bolus of ^13^C_5_-glutamine over 1 min followed by a continuous ^13^C_5_-glutamine infusion. **c**, **d** Enrichment in ^13^C-glutamine (%) from independent samples for each tumor subtype (*n* = 4 to 10) after 300 min in the liver (**c**) and control cerebral hemisphere (**d**)

To further explore glutamine metabolism in vivo, tumor glutamine uptake and metabolism were finely investigated through ^13^C enrichment analysis after [U-^13^C]glutamine infusion. After human primary tumor cells implantation into the striatum of NSG mice (Fig. 3a), tumor mass expansion was followed over time using MRI. When the tumor reached 3 mm, the mice were infused with [U-^13^C]glutamine as a bolus over 1 min followed by a continuous 5-h infusion. A time course was performed to establish maximal ^13^C-glutamine enrichment in the plasma of HOT-bearing mice. For all infused mice, 40% or more of the plasma glutamine was labeled after 60 min and this level was maintained for the duration of the infusion (Fig. 3b). At the end of the infusion, mice were sacrificed and both the liver and the brain were rapidly removed. Analysis of metabolites extracted from the liver at 300 min showed in most mice a 20% enrichment of ^13^C-glutamine (Fig. 3c). The tumor and the contralateral healthy tissue were isolated from the brain to analyze labeled glutamine and glutamine-derived metabolites. On average, an enrichment of ^13^C-glutamine of 3.6 ± 0.5% was measured in CTR tissues without any noticeable differences between HOT molecular signatures (Fig. 3d).

**Fig. 4:**
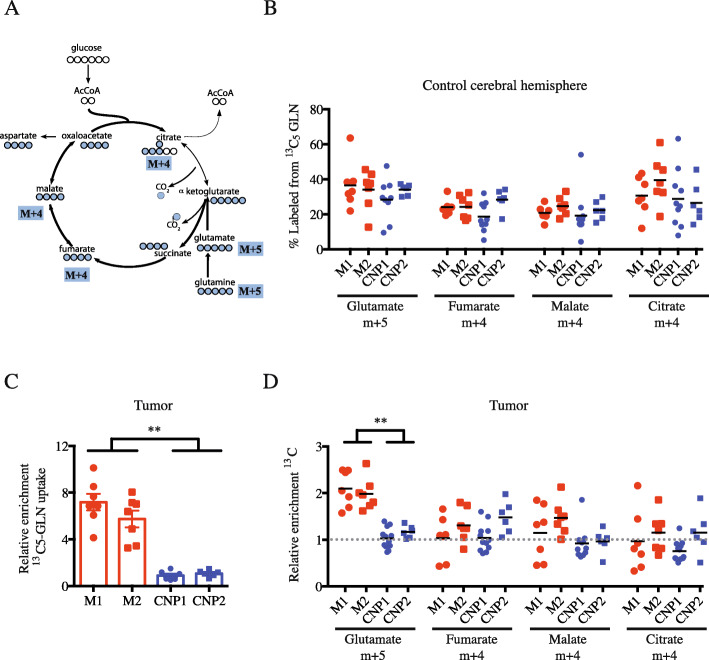
Uptake and metabolism of ^13^C-glutamine in HOT compared to control brain. **a** Schematic of labeled ^13^C-glutamine metabolism in the mitochondria. **b** Relative enrichment in the control cerebral hemisphere from HOT-bearing mice derived from each parental primary culture (CNP1, CNP2, M1, M2; samples from at least 5 independent samples) of labeled glutamate (m + 5), fumarate (m + 4), malate (m + 4), and citrate (m + 4) from ^13^C-glutamine. **c** Relative enrichment of ^13^C-glutamine uptake compared to CTR brain (%) from independent mouse HOT (*n* > 5) derived from each parental primary culture (M1, M2, CNP1, CNP2). **d** Relative enrichment compared to CTR brain of labeled glutamate, fumarate, malate, and citrate from ^13^C-glutamine from independent HOT-bearing mice (*n* > 5) derived from each parental primary culture (M1, M2, CNP1, CNP2). Two-way ANOVA, *p < 0.05; **p < 0.01

To assess glutamine metabolism in the brain, ^13^C-enrichment was examined in glutamate and several TCA cycle intermediates, and then normalized to the level of labeled glutamine in the tissue (Fig. 4a). Globally, relative ^13^C labeling on all carbons in glutamate (m + 5), fumarate (m + 4), malate (m + 4), and citrate (m + 4) reached 20 to 40% (Fig. 4b). Again, no significant differences were observed in metabolite enrichment from CTR hemispheres of HOT-bearing mice from different subtypes. We next examined ^13^C-labeled metabolites from tumors in all mice. Although absolute ^13^C enrichments were low, both mesenchymal tumors displayed enhanced enrichment relative to the control hemisphere (fold increase compared to CTR brain 7.2 ± 1.9 for M1, 5.7 ± 1.9 for M2, 0.9 ± 0.4 for CNP1, 1.1 ± 0.3 for CNP2, respectively; *p* = 0.0025) (Fig. 4c). We then examined labeling in metabolites potentially derived from ^13^C-glutamine. In CNP tumors, ^13^C-glutamine was metabolized to glutamate, fumarate, malate, and citrate in a similar manner to CTR brain (Fig. 4d). In contrast, and in agreement with increased ^13^C-glutamine uptake, the level of m + 5 glutamate was significantly increased in mesenchymal tumors as compared to CTR tissues or CNP tumors. Labeling of downstream metabolites was not significantly different between molecular subtypes or between tumors and CTR tissues. Altogether, our results provide evidence for increased glutamine uptake and conversion to glutamate in mesenchymal GBM tumors compared to CNP tumors and CTR brain.

### Inhibition of glutamine metabolism delays tumor growth in vivo

**Fig. 5:**
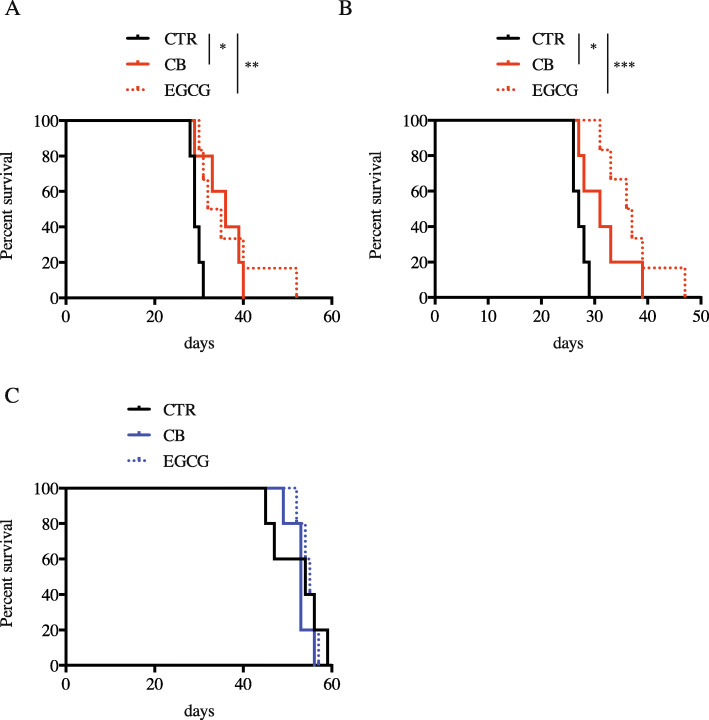
Tumor-bearing mice survival following inhibition of glutamine metabolism. **a**–**c** Mice survival following orthotopic injection of M1 (**a**), M2 (**b**), and CNP1 (**c**) primary cells. CB839 (CB, 20 μM) was injected orthotopically in the mice brain 1 h after primary GBM cell injection. EGCG (110 μM) was added to cell culture 6 days prior to orthotopic injection of tumor cells. Log rank test, *p < 0.05, **p < 0.01, ***p < 0.001

Finally, the efficacy of glutamine metabolism inhibition against tumor progression was investigated in HOT-bearing mice. Of note, in our models, the mesenchymal signature drove a faster tumor progression as compared to CNP, as evidenced by the difference in mice survival (M1, 29 days; M2, 27 days; CNP1, 54 days). First, GLS inhibitor CB839 was injected 2 times a week within the tumor bed of HOT-bearing mice. Interestingly, significant delay in tumor progression following CB839 treatment was observed in mesenchymal tumor-bearing mice (Fig. 5a, b). CB839 treatment did not affect the survival of CNP tumor-bearing mice (Fig. 5c). Similar experiments were performed using EGCG as a GDH inhibitor. In this case, cells were pre-treated with EGCG prior to the orthotopic brain injection of tumor cells. Again, EGCG treatment slowed down the proliferation of mesenchymal GBM cells without affecting the one of CNP cells (Fig. 5a–c).

## Discussion

Given the energy-generating and biosynthetic roles that glutamine plays in growing cells, inhibition of glutaminolysis might have the potential to effectively target cancer cells. We previously demonstrated that in vitro, GBM cells exhibit different metabolic profiles based on their molecular signature [9]. In particular, while all GBM cells used glucose to fuel their bioenergetic and biosynthetic needs, the mesenchymal GBM subtype displayed a singular dependency to glutamine in vitro. Here, we show in a biologically accurate mouse model of GBM that glutamine could be used as an anaplerotic substrate in both mesenchymal and CNP tumors. Moreover, mesenchymal GBM tumors uptake and utilize more glutamine in vivo as compared to other GBM molecular subtypes. If recent reports have shown that GBM tumors in vivo do not significantly catabolize glutamine but rather accumulate large pools of glutamine from glucose-derived carbon through GS [10, 14], molecular signatures of the used primary GBM cultures were not characterized. Since the probability to establish primary GBM cells with a mesenchymal signature from patient tumors is low (< 12% in our hands), it is possible that these studies do not include mesenchymal GBM culture. In fact, GS and CD133 were found highly expressed in those GBM reinforcing the possible lack of mesenchymal cells in their study since we showed that expression of these markers is restricted to glutamine-independent CNP culture [9, 14]. Our results are in agreement with numerous studies showing either distinct metabolic fuel choice and dependency based on distinct molecular signature [15] or glutamine uptake in GBM in vivo using PET imaging based on ^18^F-Fluoroglutamine [16]. Furthermore, several reports have shown that many tumors rely on glutamine to fuel TCA cycle in vivo, in agreement with glutamine dependency observed in corresponding in vitro models [17–19]. Reliance of mesenchymal GBM on glutamine metabolism in vivo is reinforced by the inhibition of key nodes in glutamine metabolism which retards tumor growth in our preclinical models. Thus, GBM definitively show distinct metabolic phenotypes that vary with molecular subtype.

GS and GLS, two enzymes catalyzing opposite reactions, control glutamine homeostasis. GS catalyzes the condensation of glutamate and ammonia to form glutamine whereas GLS, which exists as at least 2 isoforms, hydrolyses glutamine to glutamate and ammonia. The importance of tumor stroma in shaping tumor metabolism has been demonstrated in cancer from different origin. In fact, astrocytes express high level of GS which then can be used by surrounding GBM cells [14]. In our study, we focused on circulating glutamine fueling tumor cells. However, we cannot exclude that GBM cells, independent of their molecular signature, might also uptake and use synthetized glutamine from surrounding cells. In fact, a key metabolic dialogue for glutamine might exist both between tumor cells with distinct molecular signature, and also between cells from the microenvironment and tumor cells. Thus, glutamine prototrophy might dynamically evolve within a tumor with time depending on tumoral sublocalization, nutrient availability, and microenvironment metabolic features. Other studies have shown that IDH1 mutation [20, 21] or the sole presence of cystine [22] directly impacts glutamine dependency in different environmental contexts. Thus, genetics and microenvironment also directly impact GBM metabolic phenotypes [23, 24]. An increasing number of studies are now highlighting the importance of glutamine not only as an anaplerotic substrate but also as a proteogenic building block, a nitrogen donor, an exchanger for import of other amino acids, or even a signaling molecule [25]. Our data suggest that the increased glutamine utilization and conversion to glutamate in mesenchymal GBM cells may not be directly associated with differences in glutamine contributions to the TCA cycle but may rather supply glutathione synthesis given the larger glutathione pool in mesenchymal tumors. Further investigations are required to fully understand the consequences of glutamine metabolism inhibition on its pleiotropic effects.

Understanding the impact of molecular signatures on GBM development and its role in treatment resistance is primordial to design efficient therapies. For instance, the O^6^-methylguanine-DNA methyltransferase (MGMT) promoter methylation status is an important prognostic factor for TMZ efficacy [26]. Bevacizumab, a humanized monoclonal antibody against VEGF, has recently been included in several clinical trials and seems to improve prognosis of recurrent GBM [27, 28]. During the past decade, targeting cancer metabolism has emerged as a promising strategy for the development of selective antineoplastic agents. The potential to develop personalized metabolically targeted cancer therapies assumes that some tumors have metabolic preferences and vulnerabilities that distinguish them from normal tissue. Importantly, targeting glutamine metabolism in our GBM models reduces tumor growth. This is of particular interest since mesenchymal tumor cells are usually the most frequent tumor cell subtype at relapse [5] and are associated with the worst prognosis with high aggressiveness and resistance to therapies [29]. Thus, targeting glutamine metabolism for GBM therapy may provide opportunities to improve GBM prognosis. Clinical trials with GLS inhibitor CB839 have already given some promising results in triple-negative breast cancer and renal cell carcinoma [30]. This strategy has to be considered in combination with the Stupp protocol, actual gold-standard treatments prescribed to GBM patients. Further investigations must determine whether inhibition of glutamine metabolism impacts mesenchymal tumor cells’ sensibility to radiation and TMZ chemotherapy. However, previous studies have shown that modulation of mitochondrial metabolism might directly influence radiation sensitivity both in vitro and in preclinical models [31, 32].

## Conclusion

In conclusion, this study shows that GBM cells display distinct metabolic phenotypes according to their molecular subtype. This work might open new opportunity to reduce the aggressiveness of mesenchymal GBM cells.

## Supplementary information

**Additional file 1: Table S1.** Genetic alteration of parental primary cultures. Genetic loss and/or amplification were identified using RNAsequencing. **Figure S1.** Metabolic profile of Glucose6P-Fructose6P and Ribose5P in human orthotopic tumors (HOT) and control brain. Abundance of Glucose6P-Fructose6P (G6P-F6P) from 3 independent samples for CTR brain and each tumor subtype was analyzed by LCMS. Results are presented as mean ± sem. Results are compared to CTR brain and between groups using multiple t-test analysis (* p < 0.05). **Figure S2.** Mass spectrometry analysis of labeled ^13^C Glucose in CTR mice and tumor-beating mice. A. Labeled ^13^C-Glucose (m+3) in brain, liver and plasma. B. Labeled ^13^C-TCA metabolites from ^13^C-Glucose (m+3) in the brain of CTR mice, mesenchymal-tumor bearing mice and CNP tumor-bearing mice.

## Data Availability

The datasets used and/or analyzed during the current study are available from the corresponding author on request.

## Abbreviations

CNP subtype: Classical-neural-proneural subtype
CSC: Cancer stem cell
ECAR: Extracellular acidification rate
G6P: Glucose 6-phosphate
GBM: Glioblastoma
GDH: Glutamate dehydrogenase
GLS: Glutaminase
GS: Glutamine synthetase
GSEA: Gene Set Enrichment Analysis
HOT: Human orthotopic tumors
NSG: NOD-SCOD-gamma
OCR: Oxygen consumption rate
TCA: Tricarboxylic acid
TGM2: Transglutaminase-2
TMZ: Temozolomide
