# Supplementary material for: Glutamine uptake and utilization of human mesenchymal glioblastoma in orthotopic mouse model
Source: Cancer Metab. 2020 Aug 10;8:9. doi: 10.1186/s40170-020-00215-8 (PMC7416393; doi:10.1186/s40170-020-00215-8)
Supplement: Supplementary file 1 — Additional file 1: Table S1. Genetic alteration of parental primary cultures. Genetic loss and/or amplification were identified using RNAsequencing. Figure S1. Metabolic profile of Glucose6P-Fructose6P and Ribose5P in human orthotopic tumors (HOT) and control brain. Abundance of Glucose6P-Fructose6P (G6P-F6P) from 3 independent samples for CTR brain and each tumor subtype was analyzed by LCMS. Results are presented as mean ± sem. Results are compared to CTR brain and between groups using multiple t-test analysis (* p < 0.05). Figure S2. Mass spectrometry analysis of labeled 13C Glucose in CTR mice and tumor-beating mice. A. Labeled 13C-Glucose (m+3) in brain, liver and plasma. B. Labeled 13C-TCA metabolites from 13C-Glucose (m+3) in the brain of CTR mice, mesenchymal-tumor bearing mice and CNP tumor-bearing mice. [file 40170_2020_215_MOESM1_ESM.pdf]

	M1	M2	CNP1	CNP2
TP53	present	present	present	present
EGFR	normal	normal	amplified	normal
PDGFR	normal	normal	amplified	normal
PTEN	present	present	present	present
INK4a/ARF	deleted	deleted	deleted	deleted
IDH1	wild-type	wild-type	wild-type	wild-type
MGMT	absent	absent	absent	absent

Supplementary Table 1 Genomic alterations of primary cultures
Major genomic alterations reported in GBM analyzed using RNA sequencing analysis are presented for each primary cultures.

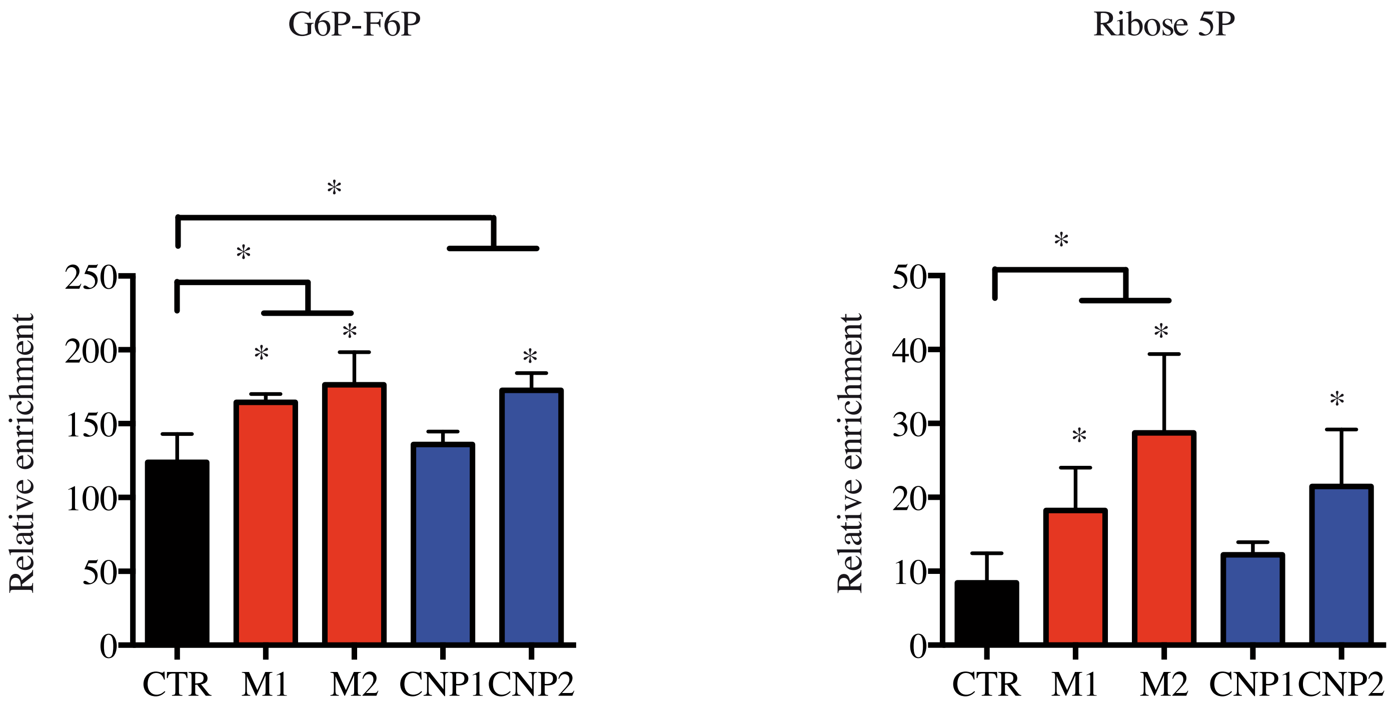

Supplementary Figure S1. Metabolic profile of Glucose6P-Fructose6P and Ribose5P in human orthotopic tumors (HOT) and control brain.
Abundance of Glucose6P-Fructose6P (G6P-F6P) from 3 independent samples for CTR brain and each tumor subtype was analyzed by LCMS. Results are presented as mean±sem. Results are compared to CTR brain and between groups using multiple t-test analysis (* $p<0.05$).

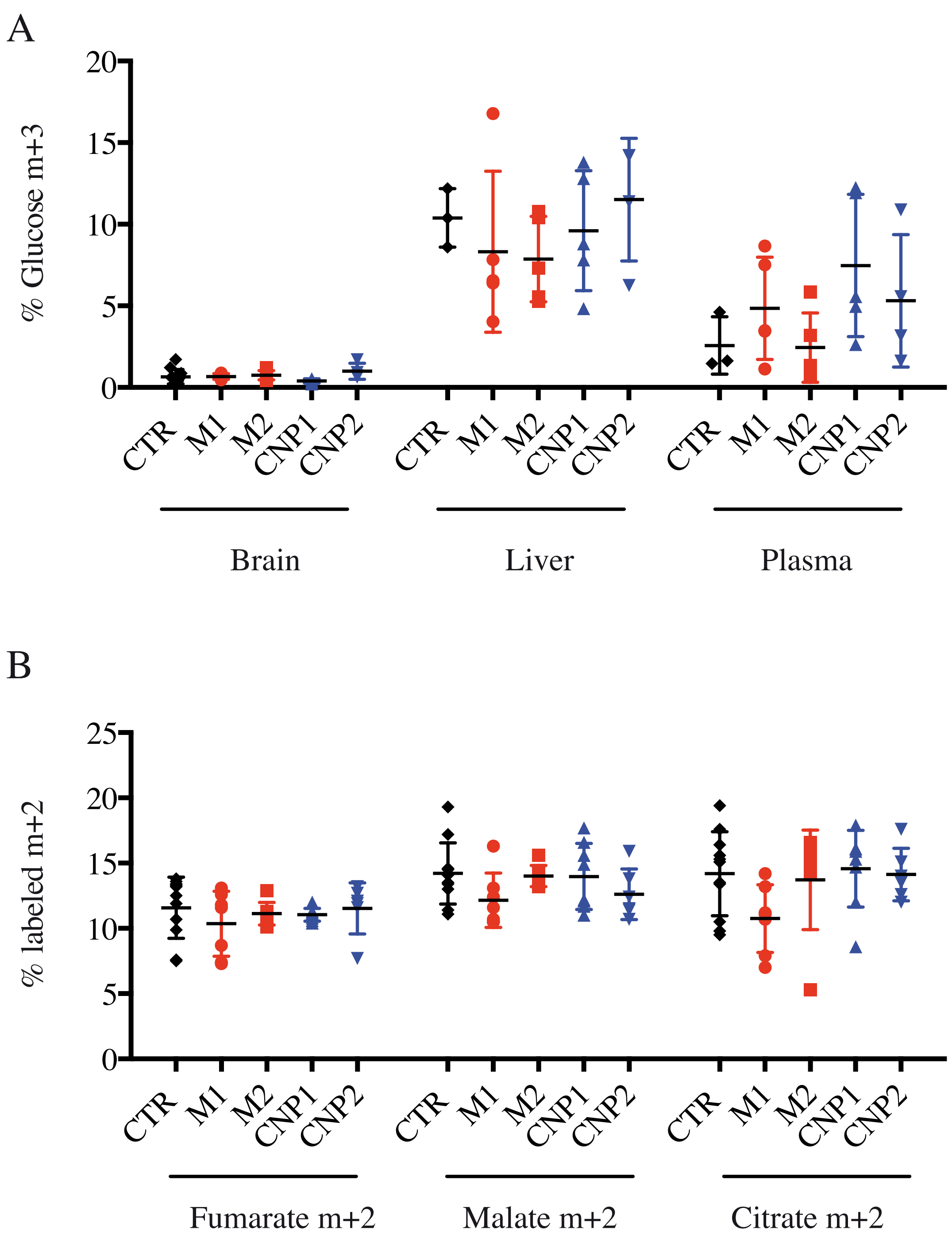

Supplementary Figure S2 Mass spectrometry analysis of labeled ^{13}C Glucose in CTR mice and tumor-bearing mice.

A. Labeled ^{13}C-Glucose (m+3) in brain, liver and plasma. B. Labeled ^{13}C-TCA metabolites from ^{13}C-Glucose (m+3) in the brain of CTR mice, mesenchymal-tumor bearing mice and CNP-tumor bearing mice.
